# Contrasting Presentations of Spigelian Hernia in a Single Surgical Unit of a Tertiary Healthcare Center: A Case Series

**DOI:** 10.7759/cureus.42238

**Published:** 2023-07-21

**Authors:** Aditya Sharma, Rahul Khanna, Ram Niwas Meena, Shashi Prakash Mishra, Panchanana Panigrahy, Seema Khanna

**Affiliations:** 1 Department of General Surgery, Institute of Medical Sciences, Banaras Hindu University, Varanasi, IND

**Keywords:** tertiary healthcare center, case report series, semilunar line, anterior abdominal wall defect, rare case of spigelian hernia

## Abstract

A Spigelian hernia is a very rare type of anterior abdominal wall hernia. The etiology revolves around the dehiscence of the transverse abdominis and internal oblique aponeurosis. The majority of Spigelian hernias develop in the lower abdomen where the posterior sheath is absent. This condition is also known as a spontaneous lateral ventral hernia or hernia of the semilunar line. It is mostly asymptomatic and is generally proven by radiological diagnosis. In the worst-case scenario, strangulation of the Spigelian hernia can occur. Here, we report a case series of contrasting presentations of Spigelian hernia in a single surgical unit of a tertiary healthcare center, considering the rarity and associated complications of Spigelian hernia.

## Introduction

A Spigelian hernia can be defined as a herniation or protrusion of preperitoneal fat or the peritoneal sac, with or without organs, caused by a deficiency in Spigelian aponeurosis [1]. The Spigelian aponeurosis is an inherently weaker area formed by the fusion of the aponeurotic layer between the semilunar line laterally and the rectus abdominis muscle medially [2]. The extension of linea semilunaris (Spigelii) from the costal margin was first described by Adrian van der Spieghel, a Flemish anatomist [3].

To diagnose this rare entity, a very high level of suspicion is required. Although abdominal ultrasonography (USG) may be informative in some cases, contrast-enhanced computed tomography (CT) is the mainstay for verifying the diagnosis, especially when it is uncertain [4]. Obesity, abdominal surgeries, chronic obstructive pulmonary disease, and abdominal trauma are among the risk factors [5]. While most Spigelian hernias are acquired, there have been case reports of congenital variants.

## Case presentation

Case one

A 64-year-old male presented to the outpatient department with the chief complaint of swelling in the left lower abdomen associated with pain for the past six months. The swelling became more prominent on straining, while it reduced in size on lying down.

**Figure 1:**
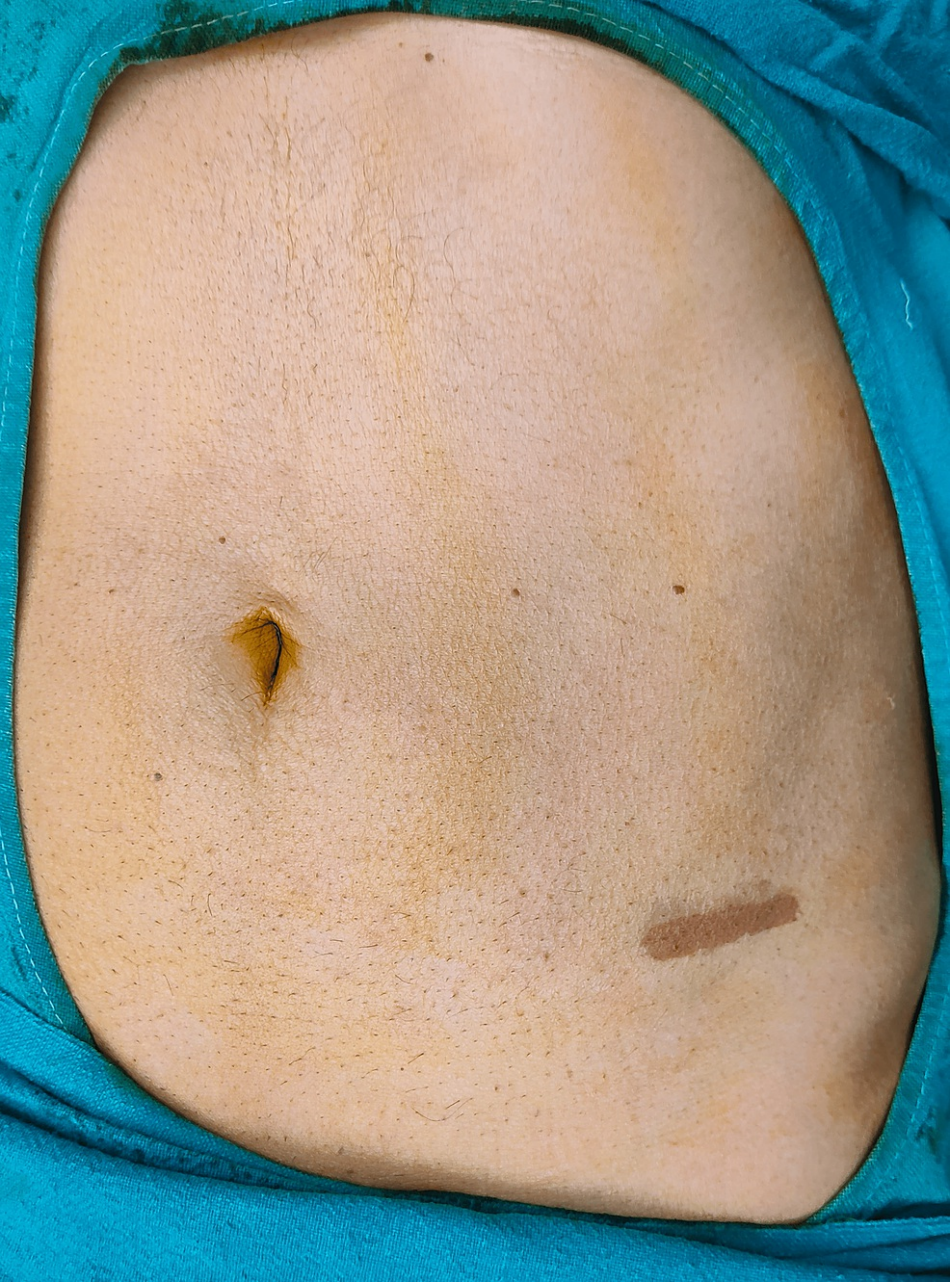
Case 1: A preoperative image of the patient showing the site of cough impulse in the left iliac region marked on the abdomen.

On examination, an oval swelling measuring 6 × 4 cm was seen in the left lumbar and iliac regions with a positive cough impulse. On palpation, a swelling measuring 6 × 4 cm was palpable in the left iliac fossa, with a smooth surface and soft consistency that could be reduced manually, as shown in Figure 1.

The hematological parameters were within normal limits. The USG of the whole abdomen suggested a defect measuring approximately 2.8 cm in the left iliac fossa with the omentum extending through the defect during the Valsalva maneuver, suggesting a Spigelian hernia. Similar findings were confirmed on CT.

**Figure 2:**
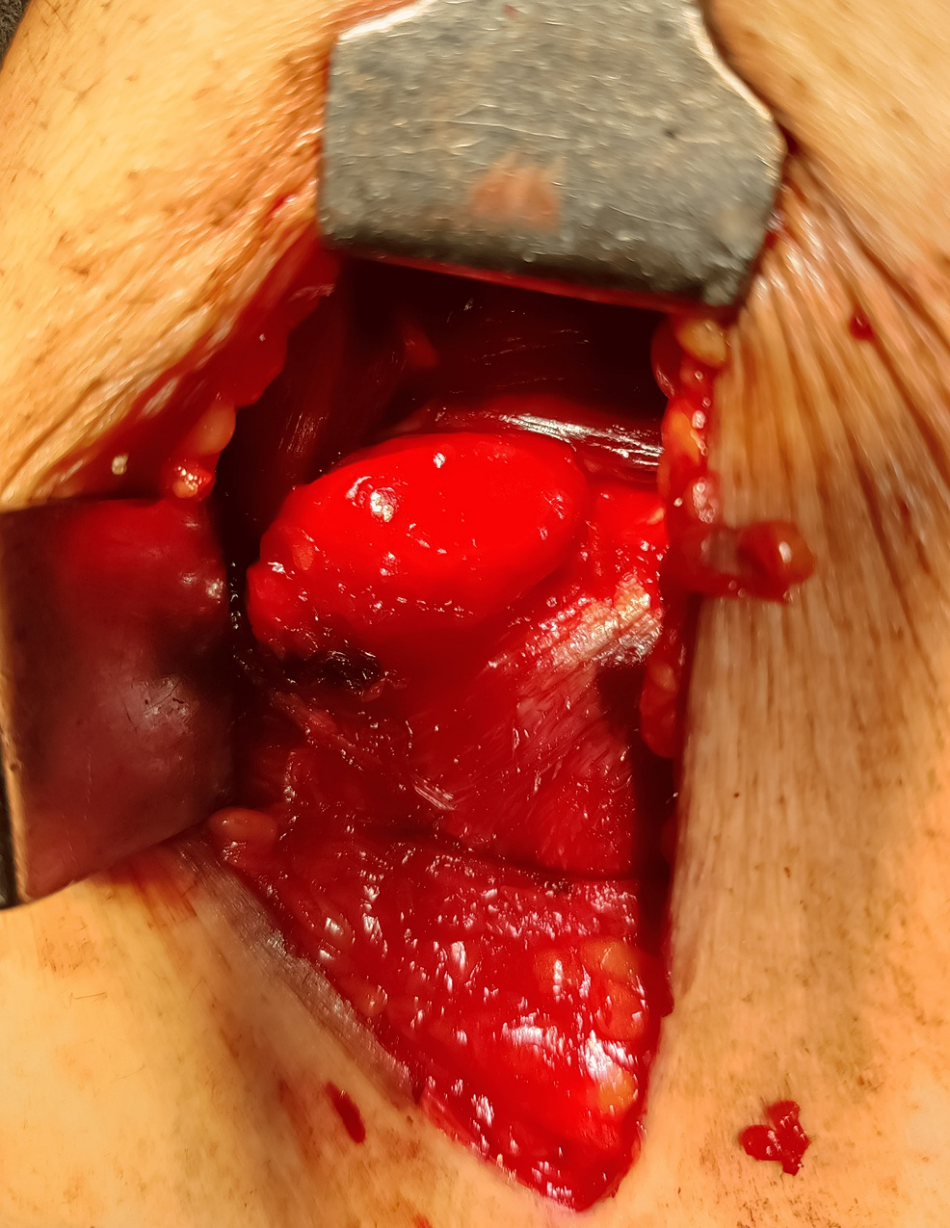
Case 1: An intraoperative picture showing a 2 × 2 cm defect at the junction of linea semilunaris and semicircularis with omentum as content.

We subjected the patient to a sublay mesh hernioplasty. Intraoperatively, a 2 × 2 cm defect was noted at the junction of linea semilunaris and semicircularis, with omentum as content, as shown in Figure 2.

The patient was commenced on oral intake on postoperative day one, which he tolerated well. The patient did well during the hospitalization period and was discharged on postoperative day two under satisfactory conditions.

Case two

A 48-year-old male presented to the outpatient department with the chief complaint of swelling in the right lower abdomen associated with a dragging sensation for the past three months. The swelling increased in size on standing, coughing, or straining and reduced in size on lying down. Additionally, it was associated with pain following meals.

On examination, a swelling measuring 8 × 5 cm was seen in the right lower abdomen, extending to the right iliac fossa. On palpation, a swelling measuring 8 × 5 cm with a 3 × 3 cm defect was present in the right lower abdomen, through which the contents could be reduced with a gurgling sound. A cough impulse was present. The margins were ill-defined, and the contents could be reduced manually. The CT of the whole abdomen, including the pelvis, suggested herniation of the descending colon and small bowel segments through the abdominal wall with a herniated sac lateral to the right rectus abdominis along the semilunar line (Spigelian line) through the transverse abdominis aponeurosis, strongly in favor of a Spigelian hernia.

The patient was posted for a sublay mesh hernioplasty, and intraoperatively, a defect measuring 3 × 3 cm was noted with omentum and bowel as contents. Primary repair of the hernia was done, and the mesh was placed between the sheath and peritoneum. Oral intake was commenced on postoperative day two, which he tolerated well. The patient did well during the hospitalization period and was discharged on postoperative day three under satisfactory conditions.

Case three

An 85-year-old male presented to the surgery emergency with the chief complaints of swelling in the left flank region for the past 10 years associated with pain in the abdomen, multiple episodes of vomiting, and absolute constipation for the past two days. As stated by the patient, he was apparently asymptomatic two days back when he developed pain in the left lower abdomen, which was sudden in onset, rapidly progressive, severe in intensity, non-radiating, aggravated by exertion, and without any relieving factors. The swelling could not be reduced by lying down or manual manipulations.

**Figure 3:**
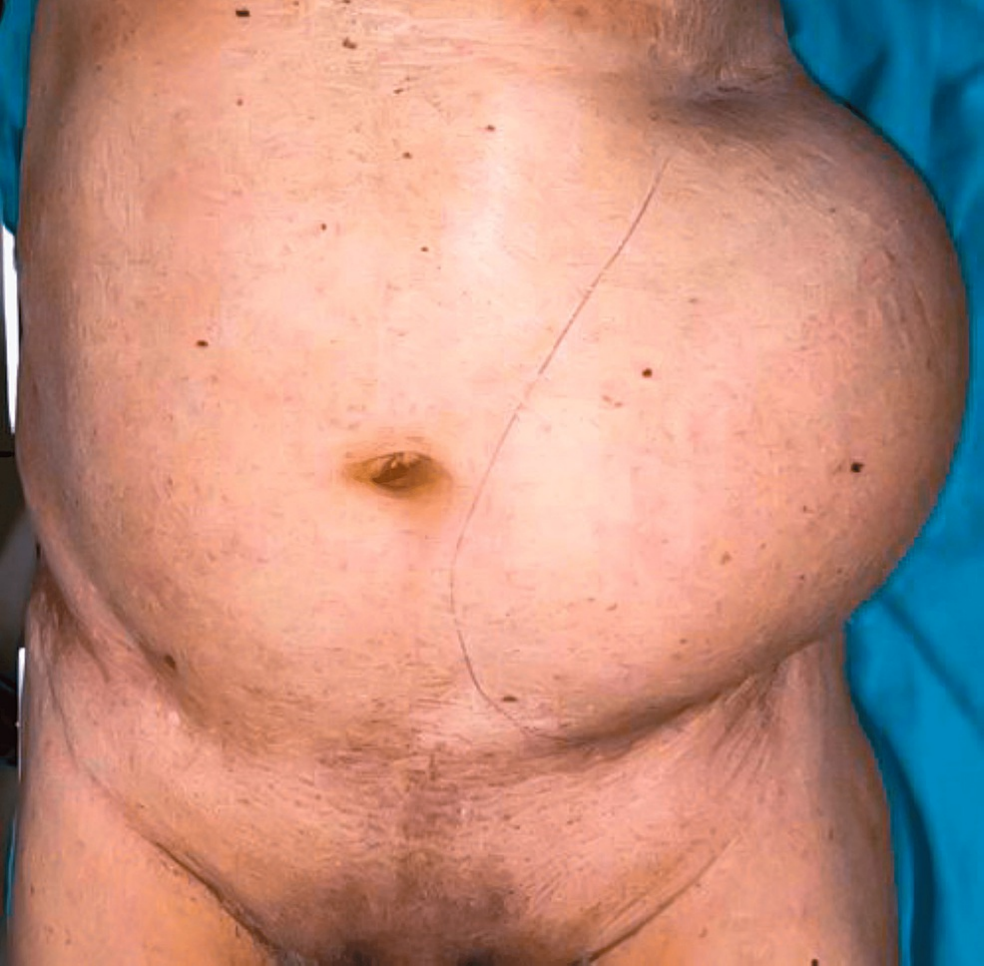
Case 3: A preoperative image of the patient showing a swelling measuring 30 × 20 cm extending from the left subcostal margin to the left iliac fossa and paraumbilical region of the abdomen.

On examination, a swelling measuring 30 × 20 cm was noted extending from the left subcostal margin to the left iliac fossa and paraumbilical region of the abdomen, as shown in Figure 3.

A cough impulse was absent. On palpation, tenderness was present, and a soft-to-firm swelling occupied the left hemi-abdomen with distinct borders and margins. The contents of the swelling could not be reduced manually. On auscultation, bowel sounds were present over the swelling. The USG of the whole abdomen suggested dilatation of small bowel loops with to and fro peristalsis and a large defect measuring 10 × 3 cm with small bowel and omentum as content, features suggestive of an obstructed Spigelian hernia. The CT was also in favor of an obstructed Spigelian hernia with small bowel and omentum as contents.

**Figure 4:**
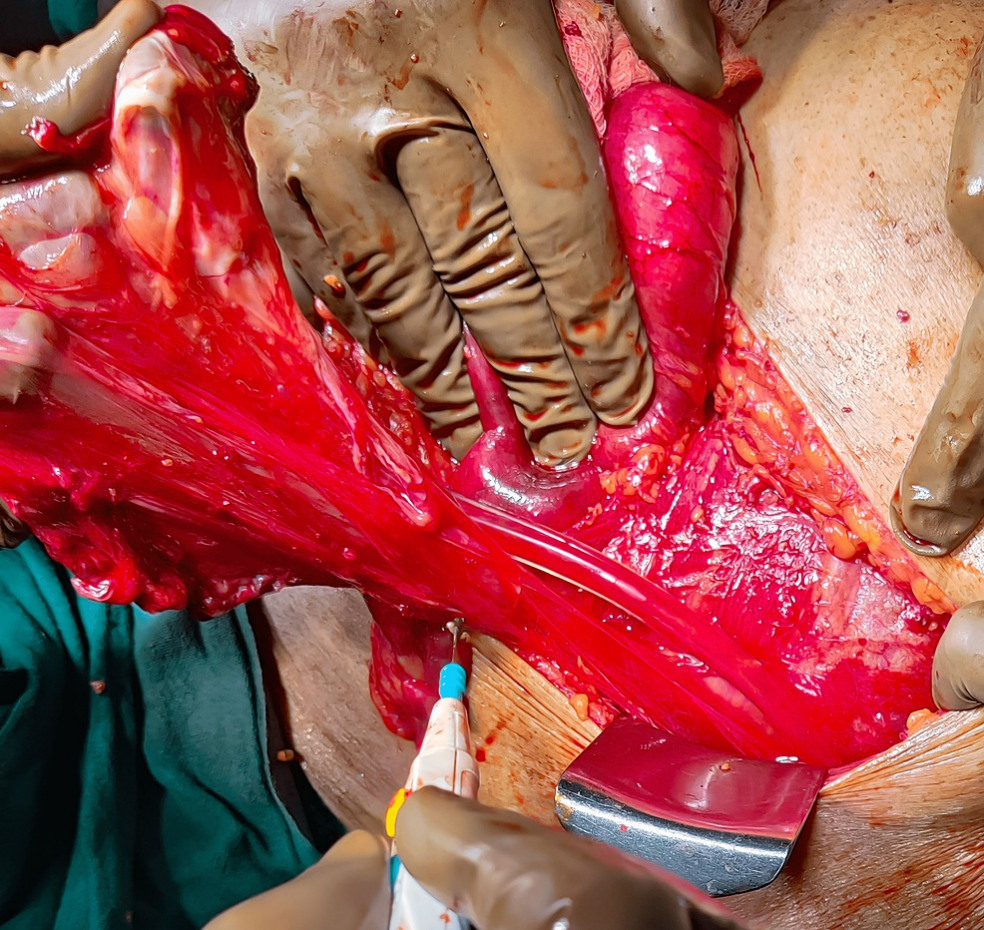
Case 3: An intraoperative image showing a 10 cm defect in the left side of the internal oblique and a 10 × 15 cm sac with small bowel and omentum as contents.

The patient was planned for mesh hernioplasty in the emergency operation theater, and during the procedure, a 10 cm defect was noted in the left side of the internal oblique, and a 10 × 15 cm sac was present with small bowel and omentum as contents, as shown in Figure 4.

The patient was started on oral intake on postoperative day five, which he tolerated well. He passed flatus on postoperative day two and stools on postoperative day five. The drain output was 40 mL serosanguinous on postoperative day one, which was reduced to minimal output on day seven, and, subsequently, the drain was removed on the same day. The patient did well during his hospital stay and was discharged on postoperative day eight under satisfactory conditions.

## Discussion

Spigelian hernias are an uncommon variant of ventral abdominal wall hernias that develop as a result of a weakness in the rectus sheath aponeurosis and transversus abdominis muscle, allowing abdominal contents to herniate through the linea semilunaris. A well-defined gap in the Spigelian aponeurosis at the Spigelian hernia belt is frequently where it begins as preperitoneal fat protruding through the defect. Nearly, 85-90% of hernias occur in this area of the aponeurosis, which is the widest region of the muscle and is located between 0 and 6 cm cephalad to the interspinous plane [6]. The typical location for the neck of the sac is in between linea semilunaris and linea semicircularis.

There are two types of Spigelian hernias, namely, acute and chronic incidental. A patient of the first type exhibits an acute abdomen and requires immediate evaluation and surgical care. In its second form, it is discovered by accident while looking into persistent and nebulous stomach pain, as in our situation.

Patients with a suspected hernia are routinely examined in erect posture in the clinical context to aid in hernia identification. The hernial sac may spread in the intramuscular plane of the abdomen and may not be well palpable. Patients with possible anterior abdominal hernias should ideally be examined in the upright position. We should ideally perform CT scans in the left and right decubitus positions instead of the supine position while elevating intra-abdominal pressure by having patients perform the Valsalva maneuver. The semi-circular line (also known as the arcuate line of Douglas) is where Spigelian hernias most frequently manifest. The Spigelian aponeurosis is a single layer and herniation-resistant below this line. According to reports, 21% of patients may exhibit small bowel obstruction as a sequela [7].

It is possible to make a preoperative clinical diagnosis in patients who have a palpable mass along the Spigelian aponeurosis, but this may be challenging in patients who have non-specific abdominal pain but no visible or palpable mass because of reducible hernia or the presence of an intramural or interparietal hernia. It is suggested to make a gridiron incision over the mass while treating a Spigelian hernia surgically. The hernial sac is made visible by cutting the external oblique aponeurosis along the direction of its fibers. Once the hernial sac is detected, it is dissected, reduced, and then the defect is closed. Thereafter, the layers of the external oblique aponeurosis and internal oblique muscle are reapproximated.

## Conclusions

Spigelian hernias should be promptly repaired surgically to prevent further complications such as bowel obstruction and strangulation, in particular, when patients present with these symptoms. Depending on experience and the accessibility of facilities for laparoscopy, either an open or laparoscopic approach may be used. In the pre-laparoscopic era, this hernia was traditionally repaired with a transverse incision and primary repair, but nowadays, these repairs are performed laparoscopically whenever possible.
